# Highly Oriented Epitaxial Hexagonal Boron Nitride Multilayers on High‐Temperature‐Resistant Single‐Crystal Aluminum Nitride (0001)

**DOI:** 10.1002/advs.202509354

**Published:** 2025-09-29

**Authors:** Xu Yang, Markus Pristovsek, Shugo Nitta, Yoshio Honda, Akihiro Ohtake, Yoshiki Sakuma, Takanobu Hiroto, Takayuki Ishida, Michio Ikezawa, Qixin Guo, Hiroshi Amano

**Affiliations:** ^1^ Institute of Materials and Systems for Sustainability Nagoya University Nagoya 464–8601 Japan; ^2^ Research Center for Electronic and Optical Materials National Institute for Materials Science 1‐1 Namiki Tsukuba Ibaraki 305‐0044 Japan; ^3^ Research Network and Facility Service Division National Institute for Materials Science 1‐2‐1 Sengen Tsukuba Ibaraki 305‐0047 Japan; ^4^ Institute of Pure and Applied Sciences University of Tsukuba 1‐1‐1 Tennoudai Tsukuba Ibaraki 305–8571 Japan; ^5^ Department of Electrical and Electronic Engineering Saga University Honjo‐1 Saga 840–8502 Japan

**Keywords:** epitaxy and annealing, hexagonal boron nitride, high temperature, multilayer, single‐crystal AlN

## Abstract

The epitaxy of high‐quality hexagonal boron nitride (hBN) multilayers on dielectric wafers is essential for hBN applications but remains challenging. Herein, highly‐oriented hBN multilayers grown on single‐crystal aluminum nitride (AlN)—AlN on sapphire and bulk AlN substrates—via metalorganic vapor phase epitaxy and high‐temperature annealing is reported. Hexagonal AlN (0001) not only provides a crystallographically commensurate base for hBN epitaxy but is thermally stable for hBN annealing up to 1800 °C, enabling the first instance of large‐area multilayer hBN with both superior out‐of‐plane and in‐plane alignments grown directly on dielectrics using a fully industry‐compatible approach. Elevated temperatures also reduce carbon and allow control over the separation of related single photon emission centers in hBN. These centers exhibit a record‐narrow wavelength distribution (578 ± 5 nm) with small zero‐phonon linewidths down to 1.44 meV, indicating the high uniformity of the achieved multilayer hBN films. This work paves an industry‐compatible way toward producing highly‐oriented homogeneous hBN multilayers on dielectrics, promising for future device and integration applications.

## Graphical abstract

High‐temperature‐resistant single crystals of aluminum nitride works best for multilayer hexagonal boron nitride (hBN) epitaxy and annealing on large‐area dielectric wafers, promising for large‐scale integration and device applications of hBN.

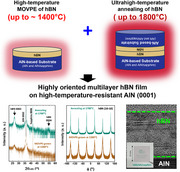

## 1 Introduction

Layered hexagonal boron nitride (hBN) is a 2D material with a very wide bandgap and deep well‐isolated states, which has been used for next‐generation advanced technologies, such as 2D electronics, room‐temperature single‐photon emission, and neutron detection.^[^ ^1^, ^2^, ^3^, ^4^, ^5^, ^6^, ^7^ ^]^ Compared with monolayer hBN, multilayer hBN serves more effectively as a substrate and dielectric layer in fabricating 2D semiconductor transistors and favors stabilizing quantum emission in hBN. Since only small hBN flakes can be mechanically exfoliated from bulk crystals, research has focused on epitaxial growth of hBN using large‐area substrates. Chemical vapor deposition (CVD) of 2D layered BN on metals has been extensively studied. However, the catalytic action only works for the first layer and limits hBN films to one or a few monolayers at most.^[^ ^8^, ^9^, ^10^, ^11^, ^12^, ^13^ ^]^ Moreover, device fabrication and characterization often demand nonmetal substrates. In such cases, an extra transfer process is required that inevitably introduces contamination and damage. Consequently, direct growth of high‐quality, large‐area hBN multilayers on microfabrication‐compatible dielectric substrates such as sapphire remains highly desirable for broader applications and integration.^[^ ^14^ ^]^ Unfortunately, dielectric wafers, such as sapphire and Si, are catalytically inactive for hBN growth. High temperatures (>1500 °C) have been suggested to improve the epitaxial quality of hBN.^[^ ^15^ ^]^ However, silicon has a melting point of ≈1410 °C, while sapphire begins to decompose above 1400–1500 °C.^[^ ^16^ ^]^ Though direct synthesis of hBN on sapphire above these temperatures via epitaxy and annealing is possible, sapphire degradation has led to uncontrollable step‐bunching and increased roughness on the surface.^[^ ^17^, ^18^ ^]^ Furthermore, a significant increase in oxygen impurities, stemming from the thermally degraded sapphire substrate, has been clearly observed in hBN grown at 1500 °C.^[^ ^19^ ^]^ These limitations greatly hinder the use of high‐temperature synthesis process that hBN favors.^[^ ^18^, ^19^, ^20^ ^]^ Aluminum nitride (AlN) is a wurtzite III‐nitride material with a hexagonal (0001) surface resembling that of hBN. AlN bulk crystals are synthesized via physical vapor transport above 2000 °C. Even on sapphire, a sufficiently thick AlN layer (at least ≈50 nm) is stable up to 1700 °C,^[^ ^21^, ^22^ ^]^ stabilizing the underlying sapphire and mitigating the up‐diffusion of oxygen from sapphire. Free‐standing AlN substrates are expected to remain stable at even higher temperatures. However, prior limited attempts to grow layered BN on relevantly thick AlN (0001) layers on foreign substrates have shown inferior lattice alignments.^[^ ^23^, ^24^, ^25^ ^]^

Herein, we report the successful synthesis of highly‐oriented epitaxial hBN multilayers on single‐crystal AlN (0001) surfaces, either relevantly thick AlN on sapphire or bulk AlN substrates, via a fully industry‐compatible approach, i.e., high‐temperature metalorganic vapor phase epitaxy (MOVPE) and subsequent annealing at higher temperatures up to 1800 °C. This developed process, which leverages high temperature (up to 1800 °C) and high‐temperature‐resistant AlN as a substrate in conjunction with optimized MOVPE growth, results in significant defect annihilation, superior lattice alignment, and relevant quantum emission in hBN. This scalable and well‐controllable approach for directly growing highly‐oriented hBN multilayer films on dielectric substrates paves the way toward emerging hBN‐based electronic and photonic applications.

## 2 Results and Discussion

### 2.1 MOVPE of hBN Multilayers on AlN and Post‐Growth Annealing Control

**Figure 1:**
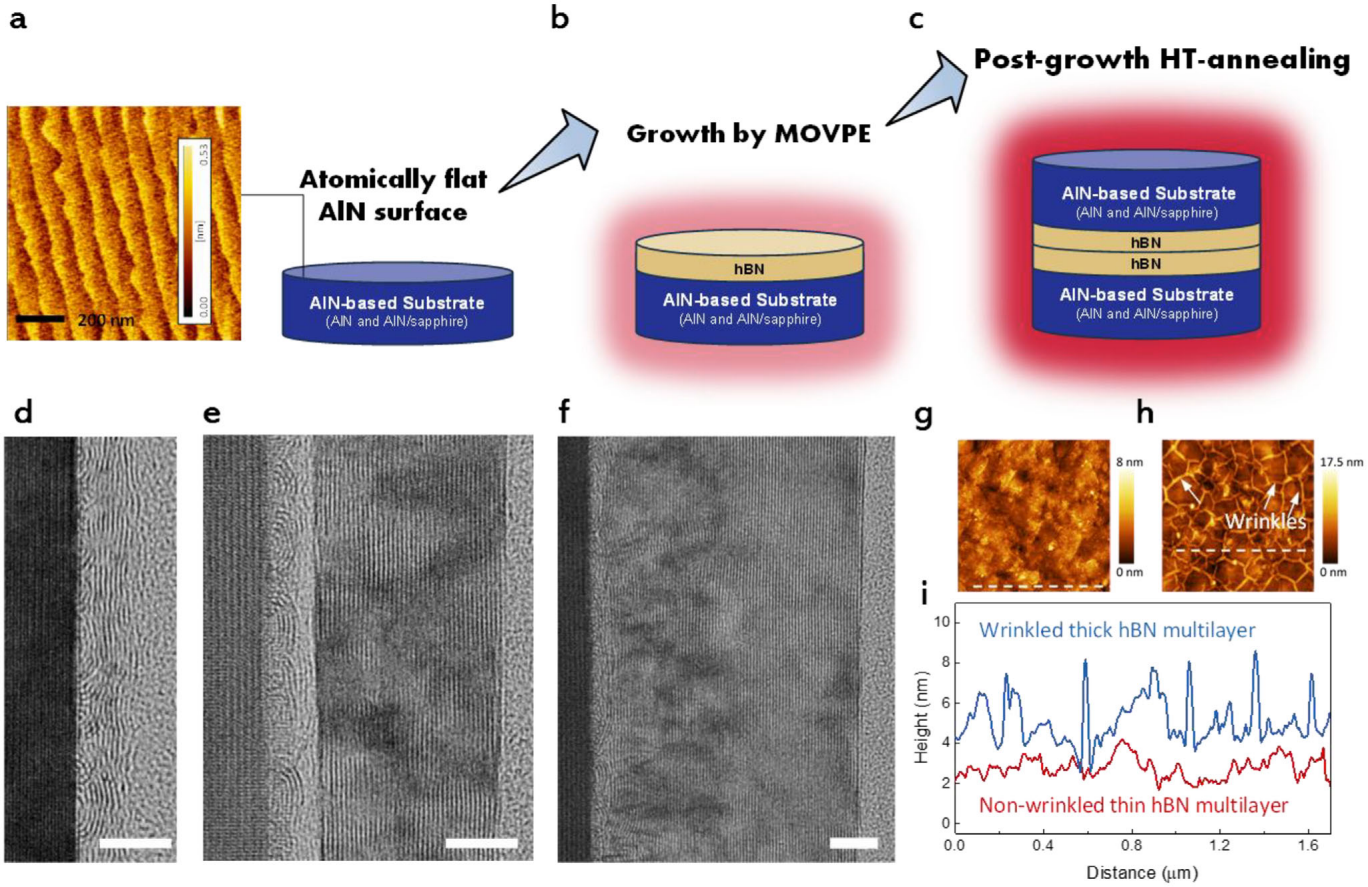
Synthesis of epitaxial hBN multilayers on single‐crystal AlN (0001) by MOVPE and high‐temperature (HT)‐annealing. a–c) Schematic illustrations depicting the synthesis process of multilayer hBN epilayers on atomically flat single‐crystal AlN‐based substrates. The AFM image in (a) shows a representative smooth AlN surface with a step‐like feature. d–f) Cross‐sectional TEM images of ≈1.5‐, 12‐ and 29‐nm‐thick 2D layered hBN films grown on AlN templates, respectively. BN at the AlN/hBN interface features dome‐like buckling growth. Scale bar: 5 nm. g,h) AFM images of (g) a nearly non‐wrinkled thin hBN multilayer film (≈1.5 nm) and (h) a wrinkled thick hBN multilayer film (12 nm). i) Height profiles of the ≈1.5 nm (red) and 12 nm (blue) hBN multilayer films on AlN templates, measured along the white dotted lines in (g) and (h).

Hereafter, we will call the single‐crystal AlN epilayer on *c*‐plane sapphire as “AlN template” to distinguish it from the single‐crystal bulk “AlN substrate”. **Figure** **1**a shows the prepared typical smooth AlN surface with an average surface roughness (R_a_) of 0.2 ± 0.1 nm prior to hBN epitaxy. On these flat surfaces, hBN was grown by MOVPE using a pulsed‐mode method with triethylborane (TEB) and ammonia (NH_3_) as B and N precursors (Figure 1b). Note that the optimized growth temperature of 1380 °C is limited by our MOVPE setup (maximum: ≈1400 °C), but it is still lower than the temperature favored by hBN as epitaxially grown on dielectrics without catalyst assistance.^[^ ^15^ ^]^ After MOVPE growth, the as‐grown layers were thus annealed at higher temperatures up to 1800 °C in N_2_ with a face‐to‐face configuration, as illustrated in Figure 1c (details in Experimental Section). Besides precise control of the initial MOVPE growth, combining high‐temperature annealing with thermally robust AlN as the substrate is key to obtaining highly‐oriented hBN grains.

We found that elevating the growth temperature with reduced nucleation rate is critical to achieve the initial epitaxial hBN multilayers on AlN (0001) surfaces by MOVPE. Reduced growth temperatures and increased TEB flow rates both led to degraded hBN growth, which potentially limits the quality of the resulting hBN crystal film even after subsequent high‐temperature annealing. Using optimized growth conditions, we first grew well‐defined 2D hBN layers on AlN, where the layer thickness can be modulated by adjusting the number of pulsed cycles during MOVPE growth. High‐resolution transmission electron microscopy (HR‐TEM) confirmed the *c*‐axis‐aligned layered hBN on the AlN templates with thicknesses of ≈1.5, 12, and 29 nm (Figure 1d–f). The buckling seen in the initial ≈1.5 nm of BN above the AlN surface is expected and has been observed before,^[^ ^26^ ^]^ which is due to the formation of covalent bonds between the initial hBN nuclei and AlN. These covalent bonds follow the bond orientation in AlN and thus are not immediately flat.

Both wrinkled and nearly wrinkle‐free multilayer hBN films were achieved (Figure 1g,h). Wrinkle formation is commonly ascribed to the thermal expansion mismatch between 2D layers (e.g., hBN and graphene) and the underlying substrates.^[^ ^27^, ^28^ ^]^ Thick hBN films on sapphire tend to exhibit pronounced wrinkling due to increased compressive strain,^[^ ^29^ ^]^ leading to larger surface roughness. In this work, the 12 nm thick hBN exhibited clear wrinkles with height variations of ≈1–5 nm and a R_a_ of 1.41 nm (Figure 1h,i). In contrast, the thinner 1.5–2.0 nm hBN showed minimal surface wrinkling with low height variations (0.5–1.5 nm) and a small R_a_ of 0.68 nm (Figure 1g–i). The surface roughness in the few‐layer hBN films is also affected by the initial buckling growth. Hence, smoother hBN films on AlN could be obtained by reducing the initial buckling. Lowering the growth temperature and increasing the TEB flux assisted in reducing buckling but compromised the interfacial crystallinity.

**Figure 2:**
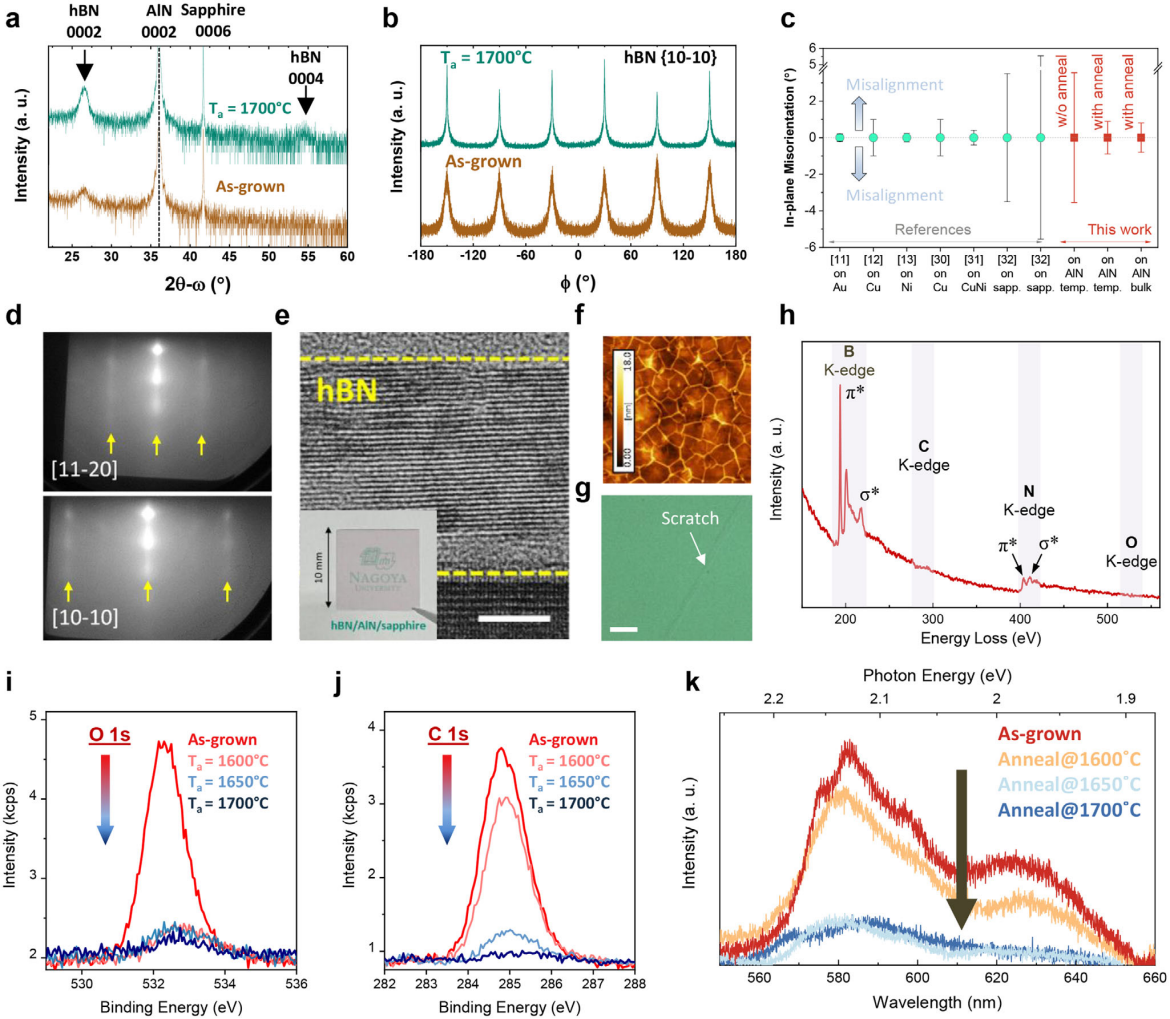
Structural and spectroscopic characterization of MOVPE‐grown and annealed multilayer hBN on AlN templates. a) Symmetric XRD 2θ‐ω measurements of as‐grown and 1700 °C annealed hBN, showing reflections from hBN 0002, hBN 0004 (after annealing) and AlN 0002 and sapphire 0006 from the AlN template. b) Corresponding in‐plane XRD ϕ measurements of hBN {10‐10} reflections for the hBN shown in (a). c) Comparison of the in‐plane misorientation of mono‐ and tri‐layers on metals and CVD‐grown hBN multilayers on sapphire from the literature with the 10‐10 in‐plane FWHM of our hBN (as‐grown and annealed hBN on AlN templates (1700 °C) and substrates (1800 °C)). d) RHEED patterns of the 1700 °C annealed hBN along the [11–20] and [10–10] directions. Yellow arrows indicate the positions of integer‐order reflections. e) Cross‐sectional TEM image of the hBN film shown in (d). Scale bar: 5 nm. Inset: Photograph of the annealed 12 nm hBN on the AlN template. f) AFM (2 × 2 µm^2^) and g) optical Nomarski microscope image of the 1700 °C annealed hBN in **(e)**; Scale bar, 10 µm. h) EELS spectrum of the TEM cross section in (e). i,j) XPS core‐levels of (i) O 1s and (j) C 1s for as‐grown and annealed hBN. k) Room‐temperature PL spectra of as‐grown and annealed hBN.

X‐ray diffraction (XRD) measures the crystallographic properties of hBN averaged over large areas but is limited by the low scattering cross‐sections of boron and nitrogen atoms. Furthermore, the symmetric 0002 reflection requires at least 4 monolayers, i.e., ≈1.3 nm hBN. Thus, it is difficult to detect the ultrathin 1.5 nm thick layered hBN by XRD. However, the XRD 2θ‐ω measurement of the 12 nm thick hBN clearly showed the 0002 reflection at 2θ = 26.6° (**Figure** **2**a), indicating *c*‐oriented hBN growth on the AlN template by MOVPE. The in‐plane *φ* measurement of the hBN 10‐10 reflection displayed six peaks appearing at 60° intervals (Figure 2b), confirming the hexagonal symmetry and high rotational alignment of the hBN multilayers. Due to the relatively low growth temperature, the reflections exhibited broad peaks with large full‐width at half maximum (FWHM) values. After annealing the hBN at 1700 °C, the 0002 reflection narrowed and increased in amplitude, and even the hBN 0004 reflection at ≈54.8° appeared (Figure 2a), indicating enhanced *c*‐axis alignment. Similarly, the hBN 10‐10 reflection in the in‐plane φ measurement narrowed from ≈7.1° for as‐grown film to ≈1.8° after annealing at 1700 °C (Figure 2b), both exceeding the XRD instrumental resolution of ≈0.2° (for the in‐plane configuration). The only comparable data in literature is the in‐plane misalignment, which gives the rotational misorientation of mostly single‐crystal monolayer hBN films relative to metal‐based substrates. Our XRD FWHMs still compare well with the reported misalignment of state‐of‐the‐art single‐crystal hBN monolayer or trilayer films grown on metals, such as Cu, Ni, and Au,^[^ ^11^, ^12^, ^13^, ^30^, ^31^ ^]^ and are much better than that of previously reported CVD‐grown hBN multilayers on sapphire (Figure 2c),^[^ ^32^ ^]^ even though our in‐plane XRD FWHM is additionally broadened by size effects and the misorientation of individual layers within the multilayer hBN. Sharp and streaky RHEED patterns observed at every 60° confirmed the high in‐plane orientation with hexagonal symmetry (Figure 2d). The stripe spacing along the [10–10] and [11–20] azimuths in the RHEED patterns yielded an in‐plane constant of ≈2.55 Å, close to the reported *a*‐lattice constant of hBN.^[^ ^33^, ^34^ ^]^ Furthermore, the coincidence of the hBN peak positions with those of AlN in the in‐plane XRD φ‐scans suggested that the epitaxial relationship is [10‐10]_hBN_//[10‐10]_AlN_ (Figure S1, Supporting Information). It has been reported that the epitaxy of layered BN on Ni can be guided by surface steps on the Ni.^[^ ^35^ ^]^ To examine the effect of AlN surface morphology, AlN templates with step‐flow and step‐bunched surfaces were prepared for hBN epitaxy. After growth, the resulting hBN films exhibited similar surface wrinkles and identical epitaxial alignment (Figure S2, Supporting Information). These results suggest that the epitaxial growth of hBN on AlN is primarily governed by the lattice symmetry of AlN and minimally influenced by its surface steps.

The microstructure of the 12 nm hBN annealed at 1700 °C on the AlN template was directly examined by cross‐sectional TEM. The TEM cross‐section in Figure 2e revealed a well‐defined *c*‐oriented layered structure that was stacked parallel to the AlN surface with an interlayer spacing of ≈3.34 Å. It is consistent with the reported value of 3.33 Å for bulk hBN.^[^ ^36^ ^]^ Compared with the as‐grown hBN multilayer (Figure 1e), the annealed hBN multilayer had a more clearly defined layered structure and fewer structural imperfections. There was no significant interface reaction between AlN and hBN, as neither the dome‐like buckling nor the surface wrinkling changed after annealing (Figure 2e,f), and the surface showed uniform contrast (Figure 2g). Thus, hBN does not react with AlN up to 1700 °C. The electron energy loss spectrum (EELS) acquired from the well‐ordered hBN cross‐section shown in Figure 2e exhibited the *π* ^*^ and *σ* ^*^ energy losses for both boron (≈190 eV) and nitrogen (≈400 eV) (Figure 2h), confirming the sp^2^ bonding configuration of hBN.^[^ ^37^ ^]^ No signals for carbon, oxygen, and aluminum were observed in the EELS spectrum after annealing at 1700 °C. Furthermore, no boron was detected in the underlying AlN (Figure S3, Supporting Information). These observations further confirm the stability of the hBN on AlN. Additionally, high‐temperature annealing reduced impurities in hBN as well. As observed by XPS, both the O 1s and C 1s core‐level intensities decreased significantly after annealing at 1650 °C and above (Figure 2i,j). Consistent with the EELS results, no Al peaks were found in the XPS survey spectrum for hBN on the AlN template after annealing at 1700 °C (Figure S4, Supporting Information). This contrasts sharply with hBN grown on sapphire, where hBN was damaged with the formation of AlN‐based compounds on the surface after annealing at 1650 °C (Figure S5, Supporting Information). As a more indirect indication, the intensity of defect luminescence decreased strongly after annealing at 1650 and 1700 °C (Figure 2k). Particularly, the emission ≈2.1 eV has been associated with carbon‐related defects in hBN.^[^ ^38^ ^]^

Recent studies have identified carbon as a key constituent of single photon emission (SPE) centers in hBN,^[^ ^39^, ^40^ ^]^ though the precise structural origin remains debatable. Compared with as‐grown hBN, the significant reduction in carbon levels after annealing enables us to resolve individual quantum emitters in the multilayer hBN even on an AlN template. Higher annealing temperatures result in fewer emission centers, as directly observed in the PL mapping measurements (Figure S6, Supporting Information). This trend aligns with the XPS C 1s core‐level data in Figure 2j, which exhibits a clear decrease in C 1s peak intensity with increasing annealing temperature. These findings indicate that the decrease in SPE centers is likely linked to the reduced carbon content in our hBN layers.

**Figure 3:**
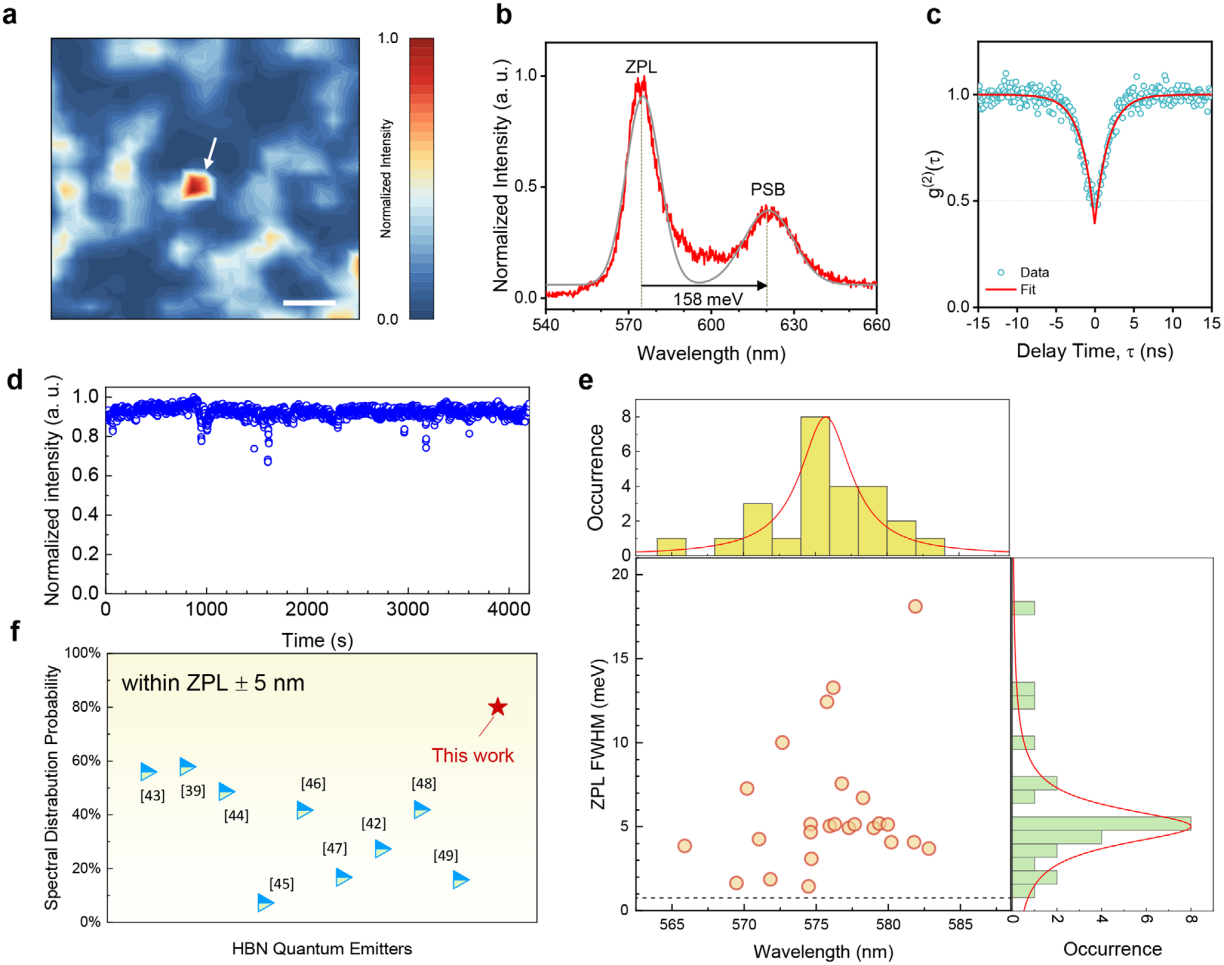
Single photon emission from annealed multilayer hBN. a) Confocal PL map showing normalized emission intensity integrated over 550–650 nm. An isolated single photon emitter, denoted as Emitter A and indicated by the white arrow, is clearly identified. Scale bar: 1 µm. b) PL spectrum (red) of Emitter A and its Gaussian‐fitted line (gray). c) Second‐order autocorrelation function g^2^(*τ*) for Emitter A (open circles) fitted with a two‐level model (red line), showing clear antibunching at zero‐time delay (g^2^(0) <0.5). d) Luminescence stability measurement of Emitter A over 70 min, showing no bleaching or blinking. e) Statistical distribution of FWHM and wavelength localization of ZPL measured at ≈5K (bin size = 2 nm). The dashed line represents the spectrometer resolution. f) Comparison of the ZPL spectral distribution window of hBN emitters obtained in this work with those of previously reported hBN emitters. All FWHM data were acquired at cryogenic temperatures.

In this study, no observable PL emission appears within the SPE wavelength range for ultrathin hBN films (4 nm or less), as shown in Figure S7a (Supporting Information). In contrast, thicker hBN films (7 nm or more) exhibit clearly detectable emissions (Figure S7b, Supporting Information). However, when the thickness becomes too large (e.g., 12 nm), the emitter density increases excessively, making it challenging to identify isolated single‐photon emitters (Figure S7c, Supporting Information). Confocal PL mapping was thus conducted to measure the emission intensity in the 550–650 nm range from a 7‐nm‐thick hBN annealed at 1700 °C, where we found a single isolated emitter within a 6 × 6 µm^2^ area (indicated by the white arrow in **Figure** **3**a). Its PL spectrum exhibited a zero‐phonon line (ZPL) at 575 nm and a phonon sideband (PSB) 158 meV below (Figure 3b), in agreement with previous studies.^[^ ^3^, ^41^ ^]^ Single photon emission was confirmed by measuring the second‐order autocorrelation function, g^(2)^(τ), using a Hanbury Brown and Twiss setup at room temperature (Figure 3c). The achieved g^(2)^(0) ≈ 0.4 indicates the quantum nature of this emitter. Time‐resolved PL measurements revealed stable fluorescence with no blinking or bleaching over 70 min for the hBN single photon emitter (Figure 3d). This is partly due to less contamination and the low defect density surrounding the emitter.

We measured ≈60 emitters from the annealed hBN multilayer at room and cryogenic temperatures under an excitation power of 100 µW. In both cases, ≈80% of the emitters exhibited ZPL wavelengths at ≈578 ± 5 nm (Figure 3e,f; Figure S8a, Supporting Information). This represents a considerably narrower spectral window compared to commonly reported visible SPE from hBN,^[^ ^39^, ^42^, ^43^, ^44^, ^45^, ^46^, ^47^, ^48^, ^49^ ^]^ highlighting the superior homogeneity of the multilayer hBN achieved in this work. As representatively shown in Figure S8b (Supporting Information), these emitters also showed a small ZPL width with a mean of 4.9 meV and a minimum of 1.44 meV measured at ≈5K. The narrow FWHMs achieved in this study are overall comparable to those reported for visible SPE from hBN grown on catalytic metals and from hBN bulk crystals,^[^ ^41^, ^42^, ^47^, ^49^, ^50^ ^]^ although ZPL width can be influenced by the excitation power used during measurement, which may vary across studies. ZPL broadening is also related to defects, i.e., charge traps, in hBN. The observed small FWHM implies a low defect density surrounding the emitters.

To get insights into carbon‐related defect candidates that may contribute to the SPE in our hBN, we compare our experimental findings with previous experimental and theoretical studies on carbon‐related defects for hBN SPE. Several defects have been theoretically proposed to account for single‐photon emission ≈2 eV, including C_B_V_N_,^[^ ^51^ ^]^ V_B_C_N_,^[^ ^39^ ^]^ and carbon trimers such as C_2_C_N_ and C_2_C_B_.^[^ ^40^, ^52^ ^]^ Among these, C_B_V_N_ and V_B_C_N_ are associated with high formation energies, several eV higher than other carbon defects like dimers and trimers.^[^ ^52^ ^]^ In contrast, C_2_C_N_ and C_2_C_B_ trimers are energetically favorable and show good agreement with the experimentally observed properties of SPE ≈2 eV. Accordingly, we align the optical properties of our SPE, including the ZPL energy (2.145 eV), PSB (158 meV), Huang–Rhys (HR) factor (1.27), and Debye–Waller (DW) factor (0.28), with the reported theoretical predications.^[^ ^52^, ^53^ ^]^ The C_2_C_N_ trimer shows excellent agreement with our results across all these parameters, including a close match in the PL line shape. Therefore, we identify the C_2_C_N_ trimer as a likely candidate to explain the observed results. However, our identification is qualitative, and further microscopic investigations are necessary to unambiguously determine the atomic structure of these emitters.

Though the presence of defects in hBN that can act as SPE centers, our high‐temperature annealed hBN still exhibited decent electrical properties. After transferring the hBN films from AlN templates onto Pt‐coated quartz substrates using a wet process, Cr/Au top electrodes were deposited to form a metal/hBN/metal sandwich‐type structure. The dielectric strength of hBN along the *c*‐axis was evaluated using these device structures. The voltage at which a sharp increase in leakage current occurs, corresponding to hard breakdown, was defined as the dielectric breakdown voltage (V_BD_). For 18 nm hBN, the Au/Cr/hBN/Pt devices exhibited a maximum V_BD_ exceeding 20 V (Figure S9, Supporting Information). The average breakdown field (E_BD_) was estimated to be 10.4 MV cm^−1^, comparable to that of hBN bulk crystals and superior to that of many previously reported CVD‐grown hBN layers, indicating the high electrical quality of our multilayer hBN films. Device‐to‐device variations in *I*–*V* characteristics were observed, which are most likely caused by wrinkles, tears, and defects in hBN that form during the non‐optimized transfer process.

### 2.2 Mechanism of Defect Healing in hBN via High Temperature Annealing

#### 2.2.1 Vacancy Migration‐Mediated Defect Healing

**Figure 4:**
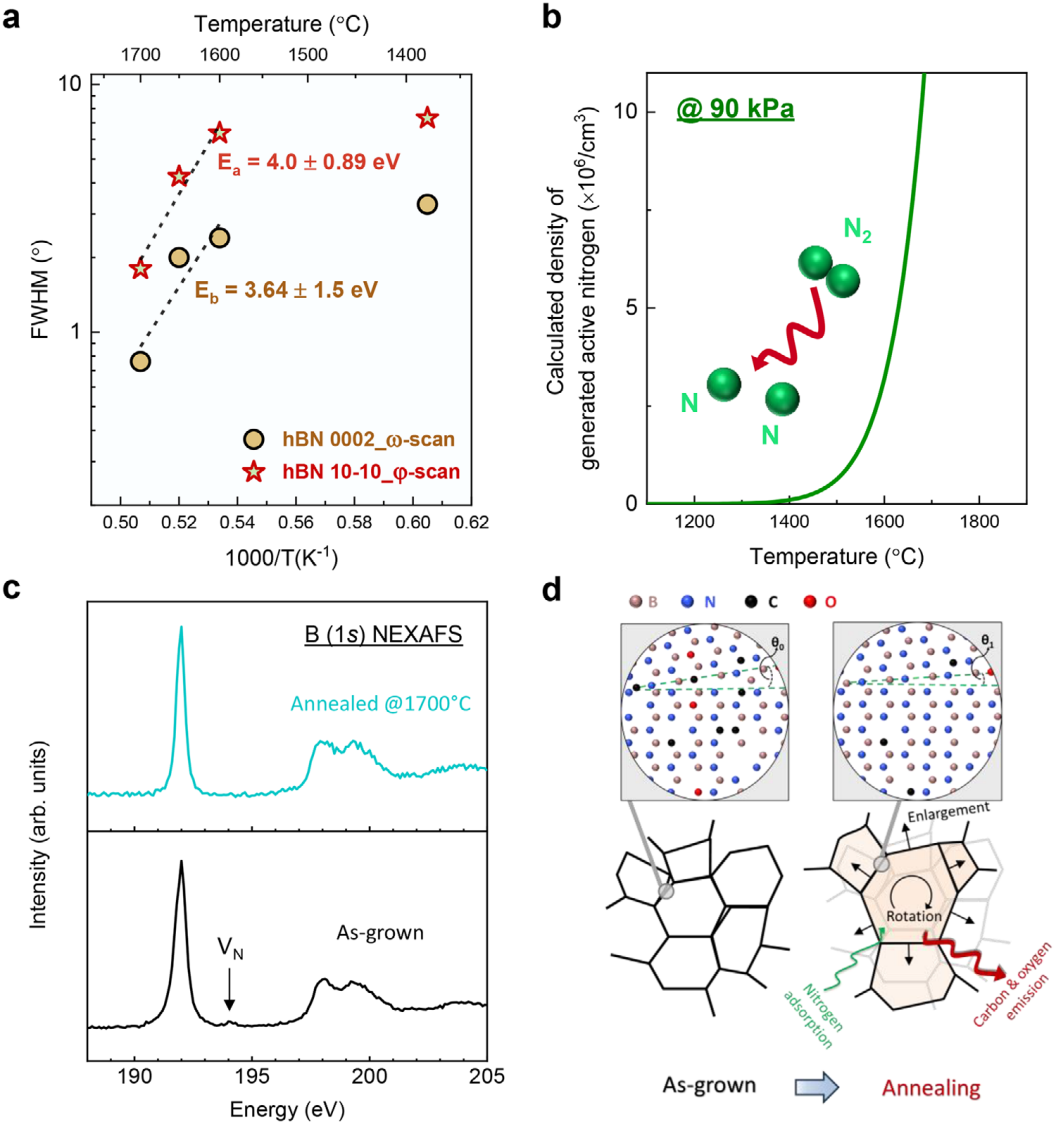
Mechanism of defect annihilation in hBN during high‐temperature annealing. a) FWHM values from ω‐scan of hBN (0002) and *φ*‐scan of hBN (10‐10) as a function of temperature. b) Active nitrogen generated from thermal dissociation of N_2_ as a function of temperature at 90 kPa. Inset: Schematic of N_2_ decomposition into active nitrogen. c) B 1*s* NEXAFS spectra of as‐grown (black) and 1700 °C annealed (green) hBN. d) Schematic illustration depicting the evolution in grains and grain boundaries of hBN before and after high‐temperature annealing. Misalignment in hBN grains: θ_x_ (x = 0, 1; θ_1_ < θ_0_).

To elucidate the microscopic processes behind the defect healing during high‐temperature annealing, we focused on the existence of defects in hBN. Commonly known defects include vacancy and impurity‐related defects (e.g., V_B_, V_N_, C_B_, C_N,_ O_B_, and O_N_).^[^ ^54^ ^]^ Recently, self‐healing induced by defect migration has been observed in graphene and conventional 3D materials.^[^ ^55^, ^56^ ^]^ In the context of hBN, the high annealing temperatures used in this study are likely to promote defect migration and annihilation in hBN, healing imperfections in grains and grain boundaries. As reported,^[^ ^54^ ^]^ an annealing temperature of 1700 °C can overcome the migration barriers of boron and nitrogen vacancies, allowing them to move through the hBN lattice and potentially be annihilated by other defects or adatoms. In addition, hBN growth can potentially form grain boundaries, which inevitably introduce various line defects incorporating different types of B–N rings, as previously reported.^[^ ^57^ ^]^ High‐temperature annealing enlarges the grain size of hBN, resulting in fewer grain boundaries, as evidenced by the enhanced crystallinity observed after annealing. Consequently, it is reasonable to expect a reduction in line defects within the hBN during the high‐temperature annealing process. By plotting the FWHMs of the hBN 0002 and 10‐10 reflections as a function of annealing temperature in **Figure** **4**a, we extracted an activation energy of ≈4.0 eV for both. This is close to the activation energies calculated by density functional theory for the migration of V_B_ (2.33–3.3 eV) and BN divacancy (V_B_‐V_N_) migration (3.0–4.5 eV).^[^ ^54^, ^58^ ^]^ This suggests that V_B_ and V_B_‐V_N_ defects migrate (and annihilate at grain boundaries) during annealing. Additionally, the bond strengths of heteroatoms in hBN, such as B─C and C─N, are weaker than the B─N bond in hBN.^[^ ^59^ ^]^ Thus, it is likely that heteroatoms like C and O move even faster than vacancies during high‐temperature annealing. Noteworthily, annealing at 1600 °C resulted in only a slight improvement relative to as‐grown hBN, whereas the FWHM strongly decreased after annealing at 1650 and 1700 °C (Figure 4a). This trend mirrors the reduction in carbon observed in XPS in Figure 2j and the decrease in PL defect signals in Figure 2k. Thus, it appears that carbon migration may be the limiting process during annealing up to 1700 °C. This also underscores the need for sufficiently high temperatures to effectively heal defects and remove impurities in hBN.

The annihilation of V_N_ and the removal of carbon may also be facilitated by active nitrogen generated by N_2_ dissociation during high‐temperature annealing. While molecular nitrogen has a high dissociation energy (≈9.76 eV),^[^ ^60^, ^61^ ^]^ thermal dissociation effectively produces atomic nitrogen above 1600–1650 °C, as calculated (Experimental Section) and plotted in Figure 4b. The resulting active nitrogen can adsorb on and diffuse into the hBN, occupying V_N_ sites and reacting with heteroatoms to form volatile molecules (e.g., CN) that then desorb from the hBN. This explains the XRD and XPS observations, which revealed significant crystallinity improvement along with a markedly reduced carbon in hBN after annealing at 1700 °C. Indeed, near‐edge X‐ray absorption fine structure (NEXAFS) measurements have indicated the removal of the nitrogen vacancy in hBN after annealing at 1700 °C (Figure 4c), evidenced by a sharper peak at 192 eV in B (1s) photoabsorption spectra and the disappearance of the peak at ≈194 eV associated with V_N_ defects.^[^ ^62^ ^]^ Consequently, in addition to vacancy migration‐mediated defect healing, the active nitrogen from thermal decomposition of N_2_ may also directly influence vacancies as well as carbon and oxygen impurities present in weakly bound grain boundaries and unstable defects. These together ultimately enhance lattice orientation and material purity by rotating and enlarging the hBN grains, as depicted in Figure 4d.

#### 2.2.2 Control of Oxygen‐Diffusion‐Induced Damage from Underlying Sapphire

Another crucial limitation arises with AlN templates from the underlying sapphire substrate. HBN (≈12 nm) grown directly on sapphire showed severe surface damage after annealing at 1650 °C (Figure S5, Supporting Information). To further determine whether the deterioration starts at the sapphire/BN interface or on the BN surface, we also annealed a thicker hBN film (≈30 nm) grown on sapphire at 1650 °C. The surface of the 30 nm hBN film on sapphire exhibited reduced damage after annealing, preserving initial surface wrinkling across most areas and forming only a few holes (Figure S10, Supporting Information). However, the XPS O 1s peak was more intense, and the Al 2p and Al 2s peaks became detectable after annealing (Figure S10, Supporting Information). These results indicate that the damage originates from the bottom, where Al and O from the decomposed sapphire diffuse into BN layer and react on the surface and at defects within the BN. Indeed, significant up‐diffusion of Al and O impurities from the sapphire substrate into the as‐grown hBN has been observed during MOVPE growth at 1380 and 1500 °C,^[^ ^19^, ^63^ ^]^ even though the direct growth of hBN on sapphire at higher temperatures is possible.^[^ ^17^, ^18^ ^]^ In contrast, hBN on AlN templates withstood annealing up to 1700 °C—more than at least 50 °C higher than the limit observed for hBN on sapphire under our experimental conditions. Thus, a sufficiently thick AlN epitaxial layer pre‐formed on the sapphire substrate effectively prevents oxygen diffusion‐induced damage to hBN. The findings above also confirm that hBN itself remains stable at these elevated temperatures.

**Figure 5:**
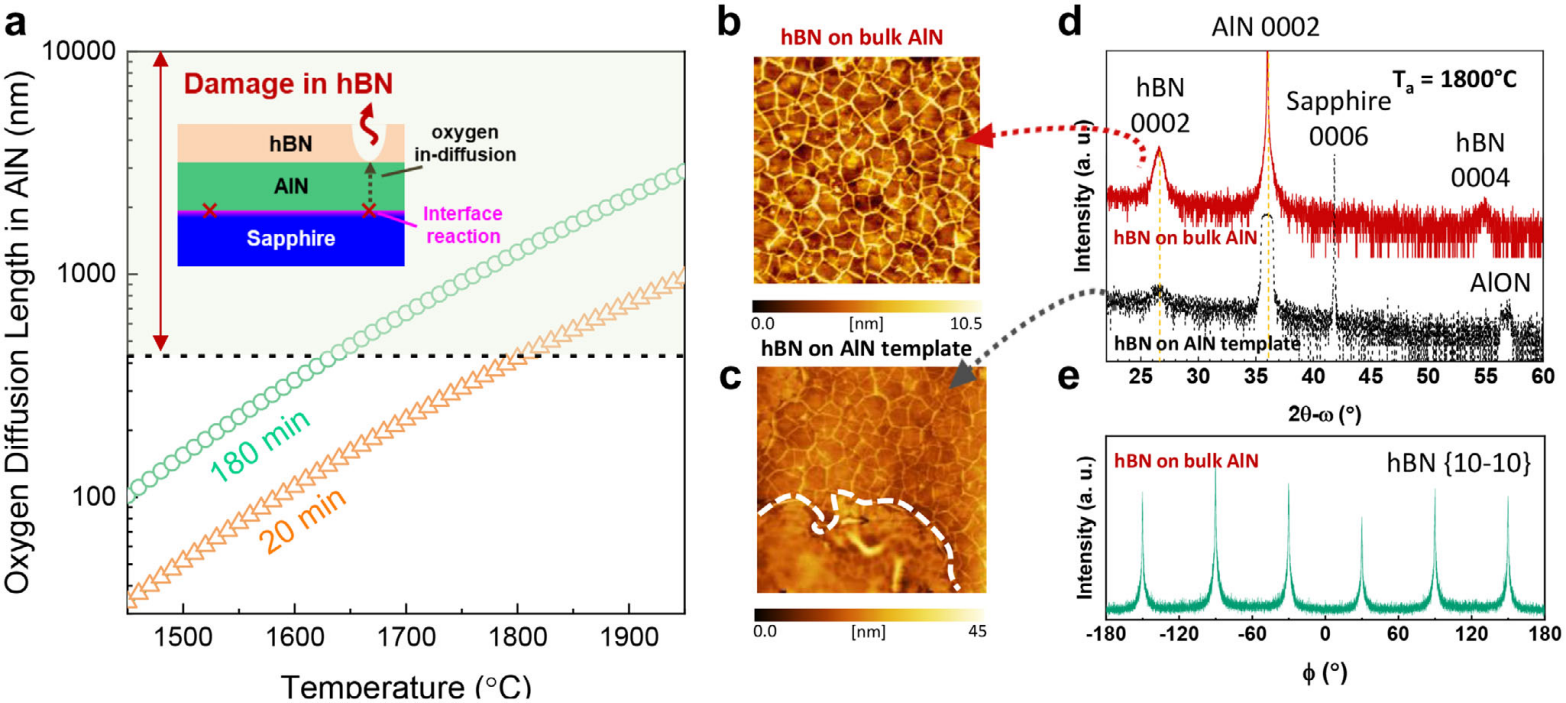
Oxygen‐diffusion‐induced degradation in annealed hBN on the AlN/sapphire template and its elimination on bulk AlN. a) Calculated diffusion length of oxygen in AlN as a function of annealing temperature and time. The dashed line indicates the typical thickness (≈430 nm) of the AlN epilayer in AlN templates used. Inset: Schematic of hBN degradation on the AlN template caused by oxygen diffusion during annealing. b,c) AFM images (5 × 5 µm^2^) of multilayer hBN after 1800 °C annealing for 20 min on (b) bulk AlN substrate and (c) AlN template. Surface damage and disappearance of wrinkles are indicated by the dashed circle. d) XRD 2θ–*ω* scans of the hBN multilayers shown in (b) and (c). e) In‐plane *φ*‐scan of the hBN {10‐10} plane for the sample on bulk AlN shown in (b).

Based on the temperature‐dependent diffusion coefficient (D) for oxygen in AlN,^[^ ^64^ ^]^ we estimated the diffusion length (λ) of oxygen in AlN using the Einstein equation $\lambda =2\sqrt{D\times t}$ with t being the annealing time. **Figure** **5**a presents the calculated λ as a function of temperature and time, where the dashed line indicates the typical thickness (≈430 nm) of the AlN epilayer pre‐formed on sapphire in this study. After 1700 °C annealing for 20 min, the oxygen diffusion length is smaller than 430 nm, indicating that oxygen cannot penetrate through the AlN layer to reach the overlying hBN. Since thermal degradation is exponential, however, higher temperatures greatly accelerate the oxidation of the AlN layer, enabling more oxygen to reach the hBN/AlN interface and then react with hBN. After 1800 °C annealing for 20 min, the AlN 0002 reflection at 2θ = 36° exhibited significant broadening, while a new reflection at 2θ ≈ 57° assigned to AlON was observed (Figure 5d). The surface wrinkling disappeared, and the crystallinity degraded (Figure 5c,d). The XPS O 1s core‐level spectrum also showed a pronounced increase in peak intensity, indicating substantial oxygen diffusion from the AlN/sapphire template into the overlying hBN layer during the 1800 °C annealing process (Figure S11, Supporting Information). Therefore, oxygen diffusion from the decomposing sapphire through the AlN during annealing ultimately sets an upper limit on AlN templates, while temperatures above ≈1600–1650 °C are needed for N_2_ dissociation and likely also for vacancy migration.^[^ ^54^ ^]^ This issue can be overcome by growing hBN directly on more stable oxygen‐free AlN bulk substrates. Notably, even after annealing at 1800 °C, the hBN surface maintained its characteristic wrinkling without damage (Figure 5b). Instead, its crystallinity was further improved relative to that annealed at 1700 °C. The X‐ray rocking curve of the hBN (0002) reflection for the sample annealed at 1800 °C exhibited a narrower FWHM of ≈0.6° (Figure S12, Supporting Information), which is smaller than that of hBN annealed at 1700 °C and lower temperatures (Figure 4a). Furthermore, Figure 2c shows that the in‐plane misalignment of hBN grown on the AlN bulk substrate and annealed at 1800 °C is less pronounced than that of hBN on the AlN template and annealed at 1700 °C. These results highlight the enhanced crystal quality of hBN achieved by higher‐temperature annealing. With further progress in nitride development, the thermally robust AlN bulk substrate may become an interesting dielectric platform for high‐quality wafer‐scale hBN epitaxy.

It is important to note that this work is fundamentally different from previous studies involving MOVPE and MBE growth of hBN on nitridated sapphire formed by intentional or unintentional surface nitridation.^[^ ^17^, ^65^ ^]^ In those cases, ultrathin layers of nominal Al_x_O_y_N_z_ or AlN (000‐1) potentially form on the sapphire surface,^[^ ^66^ ^]^ which differ markedly in chemical composition, atomic configuration, and surface properties from the single‐crystalline AlN (0001) investigated in this work. Moreover, the formation of only a few monolayers of Al_x_O_y_N_z_ or AlN on sapphire is insufficient to prevent the upward diffusion of oxygen and aluminum impurities from the degraded sapphire substrate into the hBN at elevated temperatures.

Additionally, very recent studies have demonstrated the formation of wafer‐scale single‐crystal hBN monolayers on insulating substrates using a non‐epitaxial “stamped‐like” method.^[^ ^67^, ^68^ ^]^ In this method, single‐crystalline hBN monolayers are first epitaxially grown on both sides of a Cu foil that is loosely attached to the insulator surface. After growth, the Cu foil is removed by melting or etching, leaving the as‐grown hBN layers attached to the underlying insulator. Unlike direct epitaxy on insulating substrates, this approach offers broader substrate compatibility. Since the initial monolayer hBN is epitaxially grown on catalytic Cu foils, the resulting hBN typically exhibits better crystallinity and larger grain size relative to hBN grown directly on catalyst‐free insulating substrates. However, the non‐epitaxial method still relies on the epitaxy of hBN layers on catalytic metals. A major limitation lies in thickness control because catalyst‐assisted growth on the metal surface is generally effective only for forming a single layer or, at most, a few monolayers. Indeed, the two referenced studies only demonstrated the successful preparation of monolayer hBN. Thus, achieving high‐quality multilayer hBN on insulating substrates using this method remains potentially challenging. In contrast, the approach developed in this work enables flexible control over hBN thickness, while also being industry‐compatible and readily scalable. Overall, the reported non‐epitaxial technique and the approach developed in this study can be considered complementary strategies for forming hBN layers on insulating substrates. The most suitable strategy will depend on specific application requirements, such as layer thickness, grain size, interface quality, etc.

## 3 Conclusion

In summary, we reported the successful synthesis of large‐area highly‐oriented multilayer hBN on single‐crystal AlN (0001) via high‐temperature MOVPE and subsequent annealing up to 1800 °C. High‐temperature pulsed‐mode MOVPE, which overcomes large migration barriers of boron species on the growing surface and suppresses unfavorable gas‐phase reactions between sources, ensures the initial epitaxy of hBN on AlN. Ultrahigh‐temperature annealing reduces defects via defect migration and guides the prior MOVPE‐grown hBN grains into better alignment. Combining the high‐temperature process with thermally stable AlN as a substrate is key to obtaining the epitaxial hBN multilayers on dielectric substrates with both superior out‐of‐plane and in‐plane alignments. Elevated temperatures also reduce carbon levels so that individual quantum emitters in hBN multilayers can be observed. These emitters exhibited stable room‐temperature luminescence, a narrow spectral localization at ≈578 ± 5 nm, and small ZPL widths down to 1.44 meV. Furthermore, the achieved hBN presented a dielectric breakdown strength of 10.4 MV cm^−1^, which is comparable to that of exfoliated hBN from bulk crystals. This industry‐compatible approach for scalable, high‐quality multilayer hBN fabrication on dielectric substrates is expected to accelerate the application of 2D photonics and electronics.

## 4 Experimental Section

### Preparation of High‐Quality AlN‐Based Substrate with Atomically Flat Surface

Single‐crystal AlN templates (high‐crystallinity AlN epitaxial layer on *c*‐plane sapphire) were prepared by sputtering followed by high‐temperature annealing.^[^ ^22^ ^]^ The typical thickness of the AlN epilayer is ≈430 nm with threading dislocation densities (TDs) of ≈10^8^ cm^−2^. Additionally, commercial bulk AlN substrates (HexTech, Inc.) with TDs below 10^4^ cm^−2^ were used for hBN epitaxy. Prior to growth, both templates and substrates of AlN were annealed at 1350 °C in H_2_ for 3 min to achieve a clean, atomically flat step‐like surface, as representatively shown in Figure 1a. Typically, AlN‐based substrates of ≈1 × 1 cm^2^ were used for the MOVPE growth and subsequent annealing of hBN.

### MOVPE of hBN and Post‐Growth Annealing

Multilayer hBN was grown on templates and substrates of AlN by MOVPE using TEB and NH_3_ as B and N precursors, respectively. As reported in our prior studies,^[^ ^26^, ^69^ ^]^ TEB and NH_3_ sources were alternately injected into the reactor in a pulsed‐mode growth process. Compared with simultaneous TEB and NH_3_ supply, pulsed‐mode MOVPE growth helps suppress adverse parasitic reactions between TEB and NH_3_ in the vapor phase, resulting in hBN growth with improved quality. The pulsed duration for each cycle was 2 s for TEB and 1 s for NH_3_ without interruption. The growth was carried out at 1280–1380 °C in an H_2_ atmosphere at ≈4 kPa. The flow rate of TEB ranged from 15 to 30 µmol min^−1^, while the NH_3_ flux was accordingly modulated to maintain a nominal V/III ratio of 3000. The optimized TEB flow rate and temperature for hBN growth on AlN were 15 µmol min^−1^ and 1380 °C, respectively. For comparison, hBN multilayers were also directly grown on *c*‐plane sapphire substrates using the same method. After pulsed‐mode MOVPE, the as‐grown hBN samples were annealed in a repurposed physical vapor transport system with a face‐to‐face configuration (Figure 1c), which minimizes surface damage and allows the vapor pressure of one layer to stabilize the other.^[^ ^70^ ^]^ Annealing was typically conducted in N_2_ at 90 kPa for 20 min at temperatures between 1500 and 1800 °C. All temperatures in this study were recorded by thermocouple readings.

### Characterization

Symmetric XRD measurements (2θ‐ω and ω scans) were conducted on a PANalytical X'pert five‐axis high‐resolution system using an open detector and a Cu K_α1_ source (*λ* = 1.5406 Å, 45 kV, 40 mA). In‐plane XRD experiments (φ scans) were performed using Rigaku SmartLab with a Cu Kα source (*λ* = 1.5418 Å, 45 kV, 200 mA).

Surface morphology was studied by tapping mode atomic force microscopy (NanoNavi, SII NanoTech). The crystalline quality and microstructure of hBN were examined by transmission electron microscopy (TEM, H‐9500, Hitachi) operated at 200 kV. TEM lamellas were made using a dual‐beam focused ion beam (DB‐FIB, NB5000) milling via the lift‐out method, with protective deposits of platinum, tungsten, and carbon employed during Ga ion milling. Chemical composition and fine structure were investigated by EELS using a JEOL instrument (JEMARM200F) operated at 200 kV with a ≈2 nm focal spot, which is sufficiently localized to distinguish the signal from the 2D hBN multilayers. RHEED measurements were conducted using an electron beam energy of 20 keV. As reported in the previous study,^[^ ^71^ ^]^ a well‐defined MoSe_2_ monolayer epitaxially grown on a GaAs (111) B substrate was used as the reference sample for RHEED calibration. NEXAFS measurements were carried out at the beamline BL12 at Saga Light Source in Japan using a synchrotron source with an excitation photon energy ranging from 60 to 800 eV. Chemical and bonding signatures were analyzed by XPS (Thermo Scientific) using a monochromatic Al Kα source (*hν* = 1486.7 eV). All XPS data in this study were recorded at a 90° take‐off angle using the C1s core‐level at 248.8 eV for calibration. FTIR (FTIR‐6100, JASCO) of BN was acquired in a reflection mode with a wavenumber resolution of 4 cm^−1^. All spectra were normalized to the reflectance spectrum of an aluminum mirror.

Photoluminescence (PL) measurements were carried out using a continuous‐wave 532 nm laser for excitation. PL mapping and spectra were acquired with a lab‐built confocal micro‐PL system at room and cryogenic temperatures (detection area: ≈0.8 or ≈1.6 µm), which allowed raster‐scanned PL mapping using a motorized XY stage. Spectra were recorded on a JASCO CT‐25 spectrometer equipped with a CCD (Andor DU970PC‐UVB). For low‐temperature measurements, samples were cooled in a liquid helium continuous‐flow cryostat (JANIS ST‐500) with a window leading to a lab‐built confocal microscope setup. To investigate the quantum nature of emitters, the second‐order photon correlation function g^(2)^(*τ*) was recorded at room temperature via a Hanbury‐Brown and Twiss setup consisting of a fiber‐coupled 50/50 beam splitter connected to two single‐photon counting modules. The detected events were input to a time‐correlated single‐photon counting device (TimeHarp 260 P, PicoQuant; Base resolution: 25 ps) for monitoring the second order correlation function and temporal stability of the emitter luminescence. A relatively broad 100 nm bandpass filter covering the ZPL and the PSB was used for time‐correlated single‐photon counting measurements. The second‐order autocorrelation data were fitted using a two‐level model with the following equation:

(1) $$g^{\left(2\right)}\left(\tau \right)=\;A(1-k\times \exp\left(-\frac{\left|t-t_{0}\right|}{\tau }\right)$$

where A is the uncorrelated amplitude, *k* is a pre‐factor related to single‐to‐background ratio, t_0_ is the zero‐delay offset, and *τ* is the excited state lifetime. Note that neither background subtraction nor additional spectral filtering was carried out for the autocorrelation analysis. The overall time resolution of the measurement system, ≈430 ps, also affects the measured single‐photon purity. Therefore, the actual quantum emission purity could be higher than the measured one.

### Theoretical Calculations—N_2_ Dissociation at High Temperature

Nitrogen molecules indeed could provide relevant active nitrogen through thermal dissociation at high temperatures above ≈1600–1650 °C. Assuming head‐on collisions, at least two nitrogen molecules with an energy greater than half of the dissociation energy E_d_  =  9.76 eV/2 = 4.88 eV are needed. The fraction of molecules (*N_d_*) with an energy above E_d_ can be obtained from the Maxwell–Boltzmann distribution in its energy formulation by integration from E_d_ to ∞, as noted using the following equation:

(2) $$\begin{array}{ccc} N_{d} & = & 1-\int_{0}^{E_{d}}2\sqrt{\frac{E}{\pi \left(k_{B}T\right)}}\exp\left(-\frac{E}{k_{B}T}\right)dE\\ & = & 1-[\mathrm{erf}\left(\sqrt{\frac{E_{d}}{k_{B}T}}\right)-\frac{2}{\sqrt{\pi }}\sqrt{\frac{E_{d}}{k_{B}T}}\exp\left(\frac{-E_{d}}{k_{B}T}\right)] \end{array}$$

where *k_B_* is the Boltzmann constant, *T* is the temperature. The error function can be replaced by 1, since $\mathrm{erf}(\sqrt{\frac{E_{d}}{k_{B}T}})\approx 1$ for E_d_  =  4.88 eV and the temperature span considered in this study. The number of molecules in each given value comes from the ideal gas equation:

(3) $$N=\frac{p}{k_{B}T}\;$$

The absolute number of active nitrogen (*N_nitro_*), arising from N_2_ dissociation, can thus be derived using Equation (4) by twice the product of Equation (2) and the total number of molecules at a given pressure, 90 kPa used in high‐temperature annealing in this study.

(4) $$N_{nitro}=2\;\times \;N_{d}=\frac{4}{\sqrt{\pi }}\;\sqrt{\frac{E_{d}}{k_{B}T}}\exp\left(\frac{-E_{d}}{k_{B}T}\right)\times \frac{p}{k_{B}T}\;\;$$

## Conflict of Interest

The authors declare no conflict of interest

## Supporting information



Supporting Information

## Data Availability

The data that support the findings of this study are available from the corresponding author upon reasonable request.
