# Supplementary material for: Highly Oriented Epitaxial Hexagonal Boron Nitride Multilayers on High‐Temperature‐Resistant Single‐Crystal Aluminum Nitride (0001)
Source: Adv Sci (Weinh). 2025 Sep 29;12(46):e09354. doi: 10.1002/advs.202509354 (PMC12697885; doi:10.1002/advs.202509354)
Supplement: Supplementary file 1 — Supporting Information [file ADVS-12-e09354-s001.pdf]

Supporting Information for

Highly Oriented Epitaxial Hexagonal Boron Nitride Multilayers on High-Temperature-Resistant Single-Crystal Aluminum Nitride (0001)

Xu Yang[1], Markus Pristovsek[1], Shugo Nitta[1], Yoshio Honda[1], Akihiro Ohtake[2], Yoshiki Sakuma[2], Takanobu Hiroto[3], Takayuki Ishida[4], Michio Ikezawa[4], Qixin Guo[5], and Hiroshi Amano[1]*

[1]Institute of Materials and Systems for Sustainability, Nagoya University, Nagoya, 464-8601, Japan

[2]Research Center for Electronic and Optical Materials, National Institute for Materials Science, 1-1 Namiki, Tsukuba, Ibaraki 305-0044, Japan

[3]Research Network and Facility Service Division, National Institute for Materials Science, 1-2-1 Sengen, Tsukuba, Ibaraki 305-0047, Japan

[4]Institute of Pure and Applied Sciences, University of Tsukuba, 1-1-1 Tennoudai, Tsukuba, Ibaraki 305-8571, Japan

[5]Department of Electrical and Electronic Engineering, Saga University, Honjo-1, Saga 840-8502, Japan

*Corresponding author: Xu Yang, x.yang@nagoya-u.jp

This PDF file includes:

Supplementary Text
Figs. S1 to S12

Supplementary Text

Figure S1: In-plane XRD φ-scans of the {10-10} planes of hBN and AlN for hBN grown on an AlN template and annealed at 1700 °C. Both materials exhibit six-fold rotational symmetry. The hBN is epitaxially grown on the AlN (0001) surface with the in-plane relationship of $[10\text{-}10]_{hBN}//[10\text{-}10]_{AlN}$.

Figure S2: AFM images of AlN/sapphire templates with two different surface morphologies: (a) step-bunched and (c) step-flow surfaces. Compared with the smooth, well-aligned step-flow AlN, the step-bunched AlN shows slightly larger surface roughness with wave-like steps. AFM images of MOVPE-grown hBN on (b) step-bunched AlN and (d) step-flow AlN templates. (e,f) XRD data for out-of-plane 2θ/ω - and in-plane φ-scans of hBN grown on two AlN templates shown in (a) and (c). No significant differences were observed in the surface wrinkling of hBN or in the epitaxial alignment between the as-grown hBN and AlN. The results indicate that epitaxial growth of hBN is not dominantly governed by AlN surface steps in this study.

Figure S3: EELS spectrum of the underlying AlN acquired from the TEM cross section in Fig. 2e. It shows only Al and N signals with no detectable boron, implying the absence of boron diffusion into the underlying AlN after annealing at 1700°C.

Figure S4: XPS survey of hBN (12 nm) on an AlN template after annealing at 1700°C for 20 min. There are no detectable Al-related signals (Al 2p and Al 2s peaks), which indicates that Al from the underlying AlN did not significantly diffuse into the topside hBN and that the hBN/AlN interface remains stable and intact even after 1700°C annealing. The result contrasts sharply with the observations in **Figs. S5**, which show notable Al up-diffusion from the sapphire substrate into the as-grown hBN on the top after annealing at an even lower temperature of 1650°C.

Figure S5: (a,b) AFM images and (c) FTIR spectra of 12 nm hBN on *c*-plane sapphire substrates before and after annealing at 1650°C. The surface morphology shows a distinct change before and after annealing. Although the root mean square (RMS) roughness remains unchanged, the surface wrinkles disappear after annealing. Additionally, the characteristic FTIR vibration mode of hBN at ~1368 cm^{-1} vanishes, while an AlN-related mode emerges. These observations suggest that hBN on sapphire undergoes significant degradation after annealing at 1650 °C, consistent with the AFM images shown in (a) and (b).

Figure S6: Confocal PL mapping of hBN annealed at (a) 1700°C and (c) 1650°C, showing the intensity of emitted light in the 550–650 nm range. (b) and (d) present the corresponding 3D surface plots of (a) and (c), respectively. Clearly, the emission center density for hBN annealed at 1700 °C is lower than that annealed at 1650 °C.

Figure S7: Confocal PL spectra and mappings of hBN films with different thicknesses after annealing at 1700 °C. No observable PL emission was detected near 580 nm from the 4 nm hBN film, whereas emission became visible at certain spots in the 7 nm hBN film. The PL mappings clearly showed isolated single photon emitters in the 7 nm hBN film. However, identifying individual emitters becomes difficult in the 12 nm hBN sample due to the high emitter density.

Figure S8: (a) Histogram of room-temperature ZPL wavelength distribution. The shaded area highlights the range at 578.5 ± 5 nm, which contains about 83% of the emitters and indicates a good spectral localization in the hBN emitters. (b) A representative high-resolution low-temperature PL spectrum in our hBN SPE centers, showing a narrow linewidth of ~ 1.4 meV.

Figure S9: (a) I-V curves for 18-nm-thick hBN devices measured in vacuum with an applied bias of 15 ~ 25 V in vacuum. A maximum V_{BD} exceeding 20 V is attained among the measurable devices, where leakage current sharply increased and device breakdown occurred. A few devices did not undergo hard breakdown under the applied bias used during our measurements. (b) E_{BD} for 18 nm hBN obtained in this work and its comparison with reported values in the literature[1–7]. An average E_{BD} of 10.4 MV/cm, with a maximum value exceeding 12 MV/cm, was achieved. The achieved E_{BD} is comparable to that of bulk hBN crystals and is higher than that of many CVD-grown hBN layers reported previously.

Figure S10: AFM images and XPS results of as-grown and annealed hBN films (30 nm) on sapphire substrates. The AFM images show that the characteristic surface wrinkles of the as-grown hBN film were largely maintained after annealing at 1650°C, though a few surface holes appeared. Compared with the results in **Figs. S5a and b**, the reduced surface damage in the thicker hBN film after annealing suggests that hBN itself remains stable during the annealing process and that the degradation likely initiated from the bottom interface. Unlike the as-grown hBN, the Al signal (Al 2s and Al 2p peaks) was clearly detected in the annealed hBN by XPS (**Fig. S5c**), indicating the up-diffusion of Al from the sapphire substrate during annealing at 1650°C. Furthermore, the intensity of the O 1s core-level increased for the annealed hBN on sapphire, which can be partly ascribed to enhanced oxygen incorporation from the sapphire into the hBN film.

Figure S11: XPS O 1s spectra of hBN on an AlN template before and after annealing at 1800°C.

Figure S12: X-ray rocking curve of the hBN (0002) reflection for hBN on an AlN substrate after annealing at 1800°C.

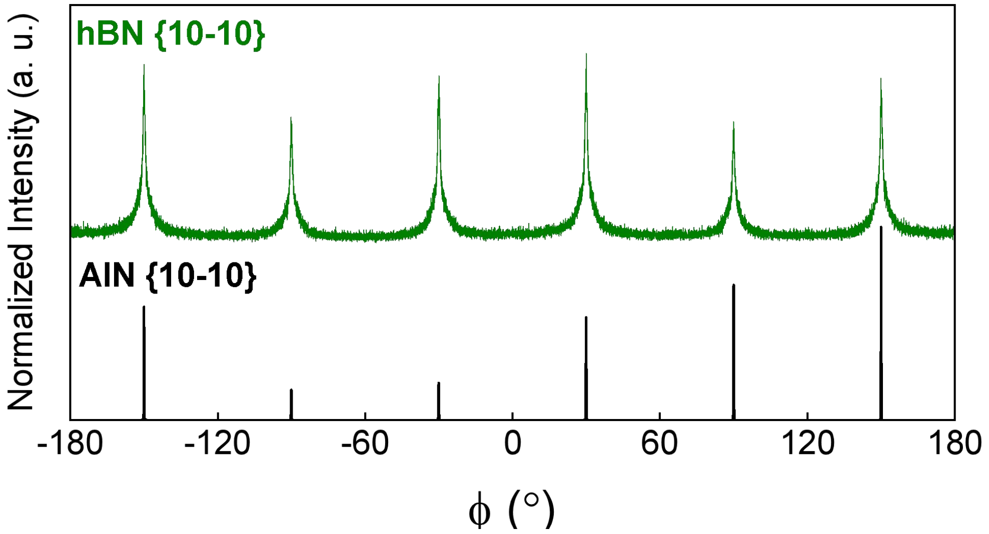

Supplementary Figure S1 | In-plane XRD φ-scans of the {10-10} planes of hBN and AlN, where the hBN grown on the AlN template and then annealed at 1700°C.

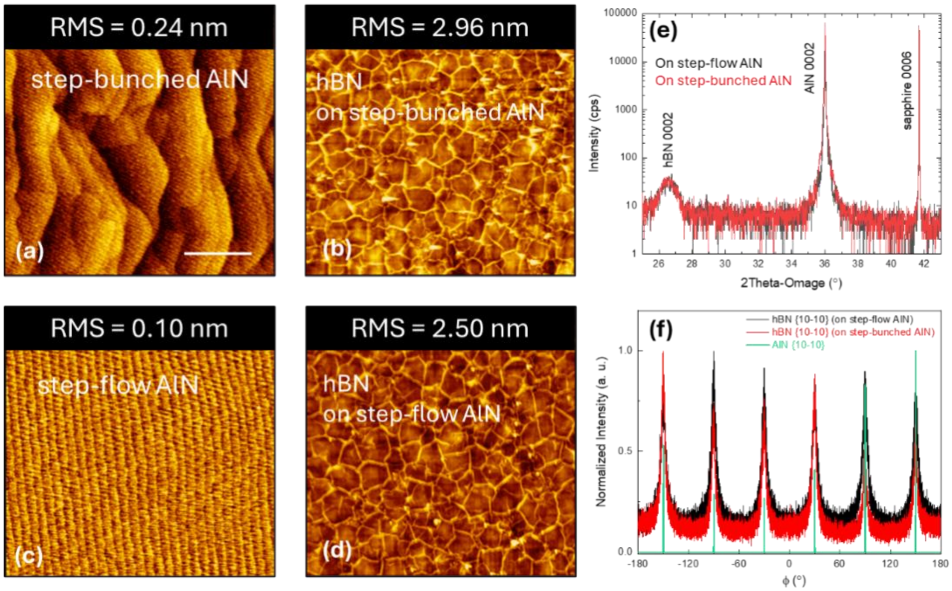

Supplementary Figure S2 | (a-d) AFM images of step-bunched and step-flow AlN and MOVPE-grown hBN films on both AlN surfaces. Scan bar: 500 nm. (e) XRD 2θ−ω measurements around the hBN (0002) reflection for the samples shown in (b) and (d). (f) In-plane XRD φ-scans of the {10-10} planes of hBN and AlN for hBN grown on AlN templates with the two different surface morphologies shown in (a) and (c).

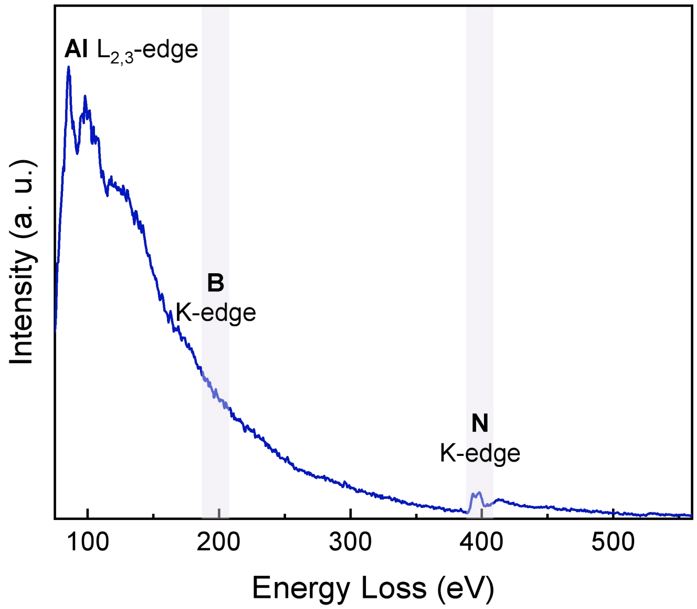

Supplementary Figure S3 | EELS spectrum of the underlying AlN from the TEM cross section shown in **Fig. 2e.** The spectrum was acquired from a location approximately 5 nm below the hBN/AlN interface. There is no observable boron signal in the underlying AlN layer.

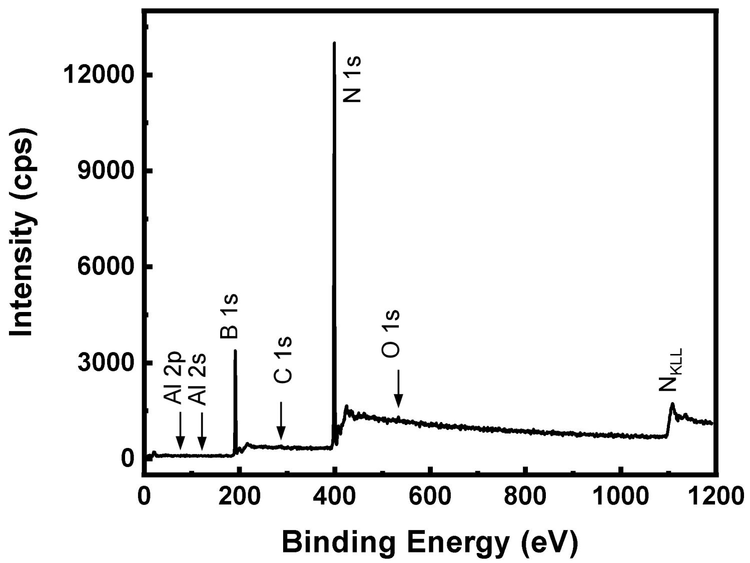

Supplementary Figure S4 | XPS survey of hBN (~ 12 nm) grown on an AlN template and annealed at 1700ºC.

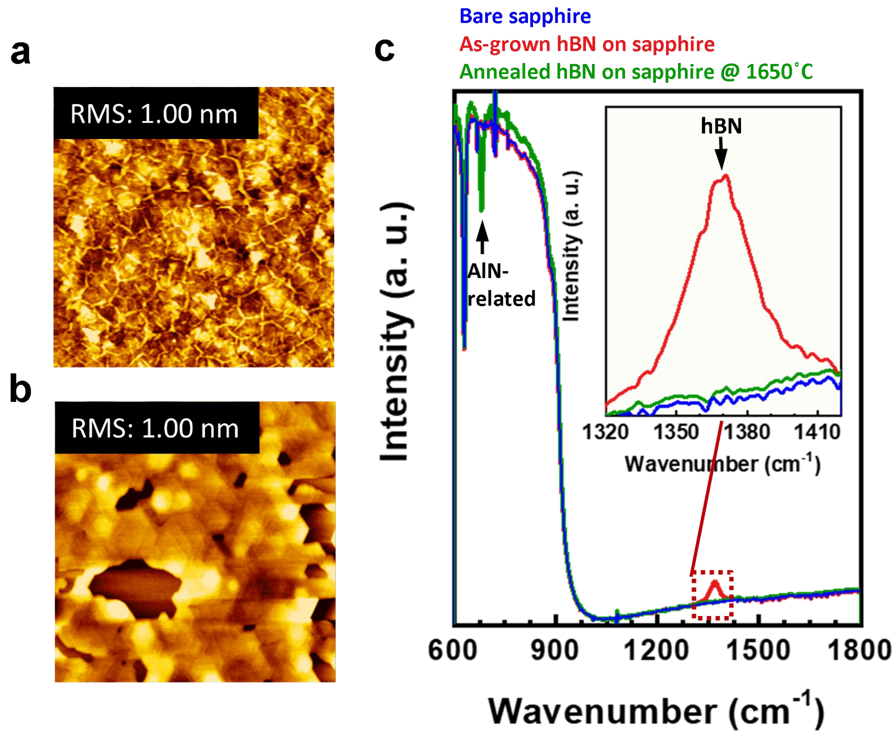

Supplementary Figure S5 | (a,b) AFM images of as-grown (top) and 1650°C annealed (bottom) hBN (~ 12 nm) directly grown on *c*-plane sapphire. Scan area: 2 × 2 μm^2. (c) Corresponding FTIR spectra of the as-grown and annealed hBN multilayers shown in (a) and (b). A reference FTIR spectrum of bare sapphire is included for comparison.

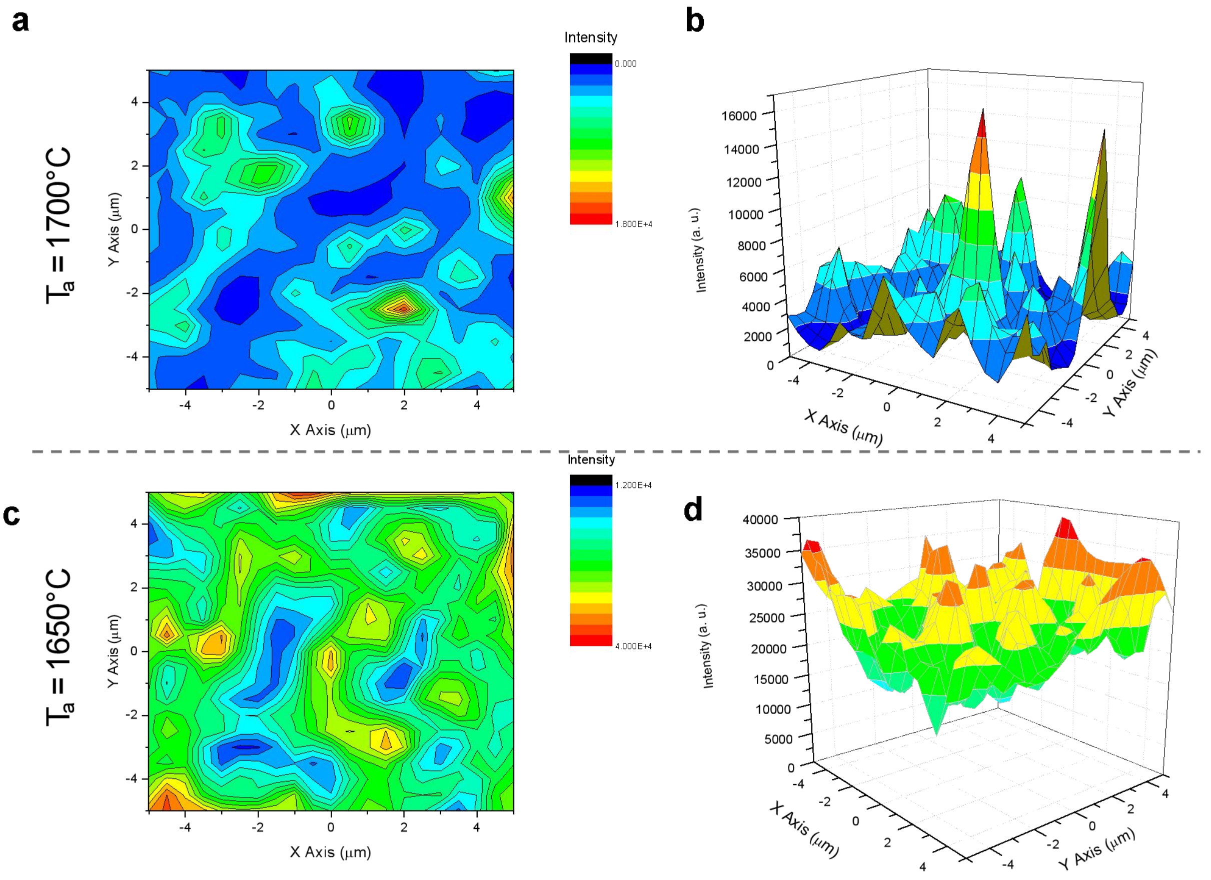

Supplementary Figure S6 | Confocal PL mapping of hBN annealed at (a) 1700°C and (c) 1650°C, which measured the intensity of emitted light in the range of 550 ~ 650 nm. (b) and (d) show the corresponding 3D maps of (a) and (c), respectively.

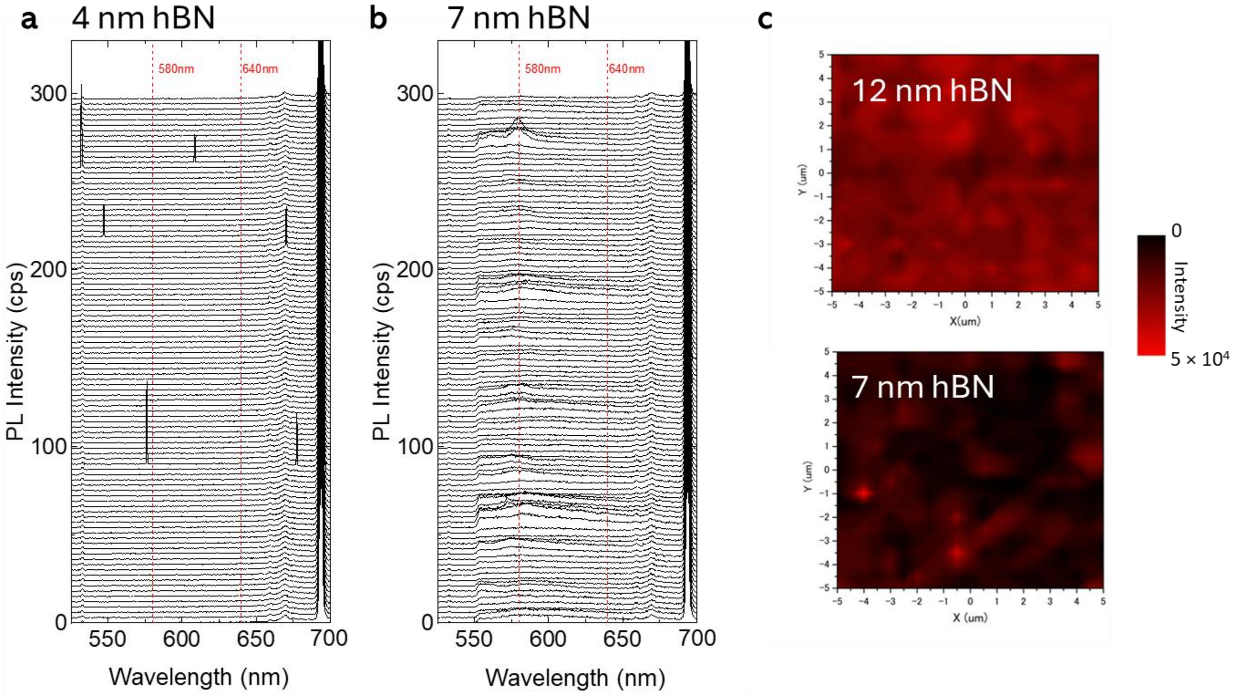

Supplementary Figure S7 | Confocal PL spectra collected from 100 spots for (a) 4 nm hBN and (b) 7 nm hBN. There is no observable PL emission around 580 nm in the 4 nm hBN, whereas emission is visible at some spots in the 7 nm hBN. (c) PL mappings of 12 nm and 7 nm hBN films, showing the intensity of emitted light in the range of 550 ~ 650 nm.

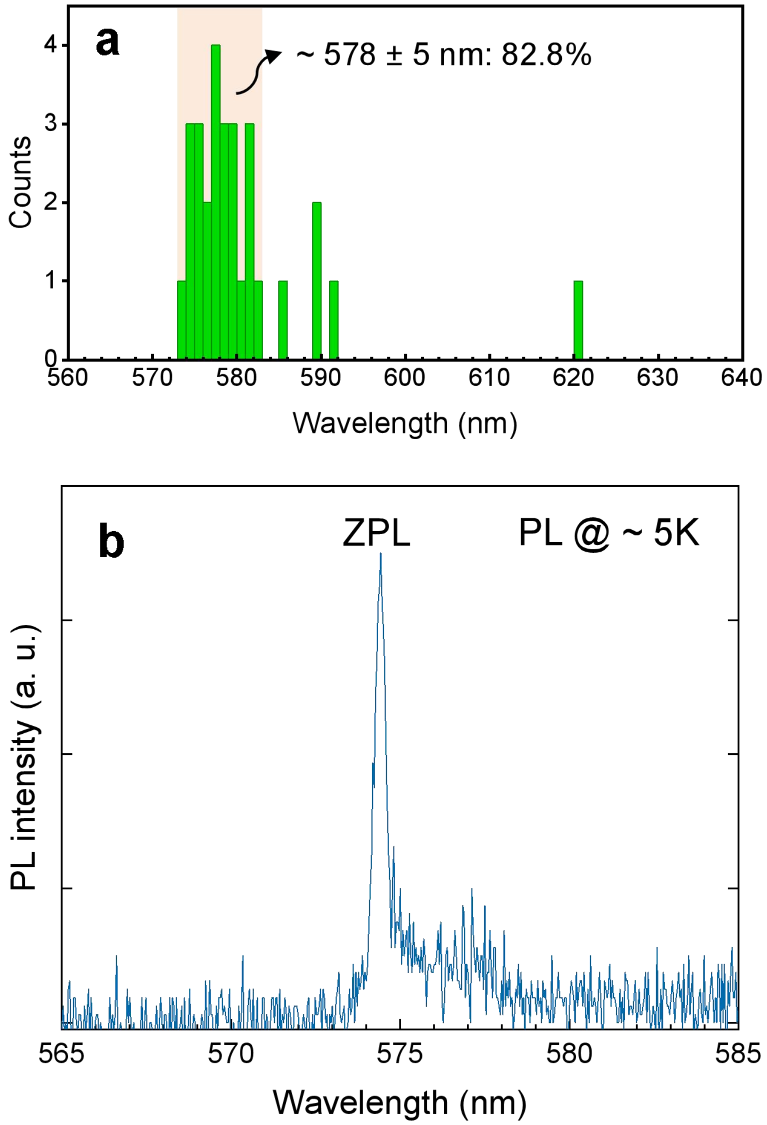

Supplementary Figure S8 | (a) ZPL wavelength distribution histogram for hBN emitters measured at room temperature (bin size = 1 nm). The shaded area highlights the 578.5 ± 5 nm range, which contains around 83% of the emitters. (b) High-resolution spectrum of the hBN emitter measured at ~ 5K.

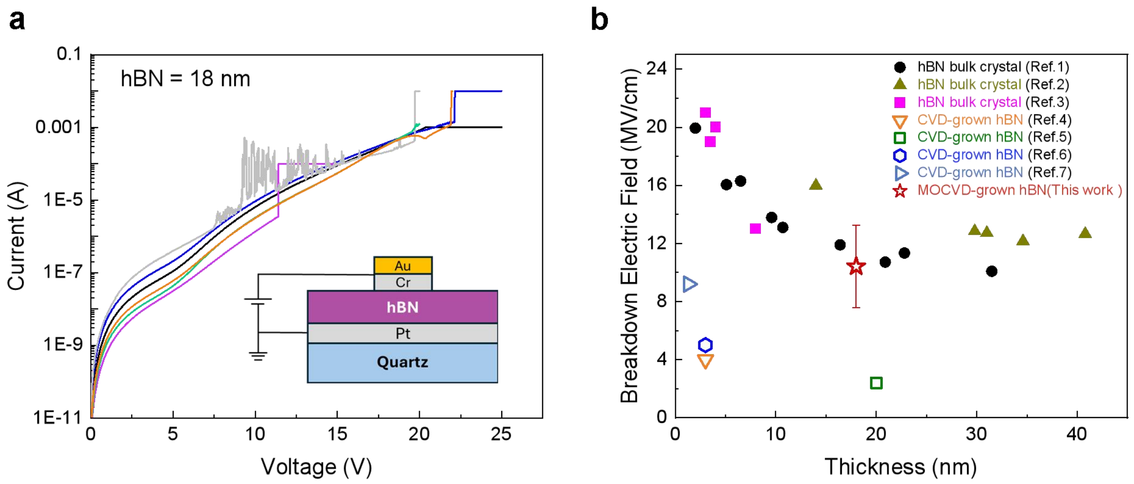

Supplementary Figure S9 | Dielectric breakdown of hBN in the direction parallel to the c axis. (a) I-V curves for hBN with a thickness of 18 nm. Inset: Schematic of the Au/Cr/hBN/Pt devices on quartz. Au/Cr electrode diameter: 200 μm. (b) E_{BD} for 18 nm hBN obtained in this study versus previously reported data for hBN films of various thickness.

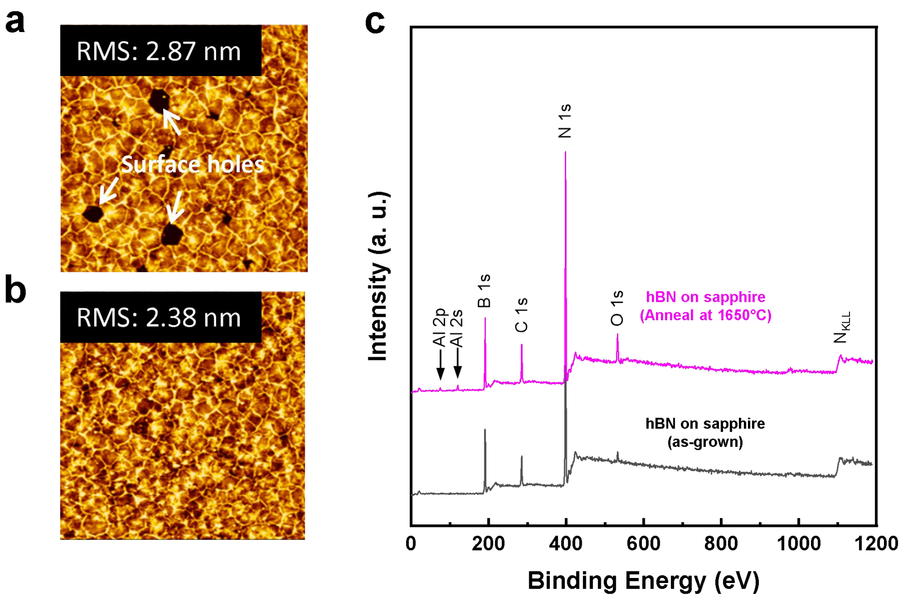

Supplementary Figure S10 | As-grown and annealed multilayer hBN films (~ 30 nm) on sapphire substrates. (a,b) AFM images of hBN films on sapphire before (bottom) and after (top) high-temperature annealing at 1650°C for 20 min. Scan area: 5 × 5 μm^2. (c) Corresponding XPS wide scans of as-grown (black) and annealed hBN on sapphire.

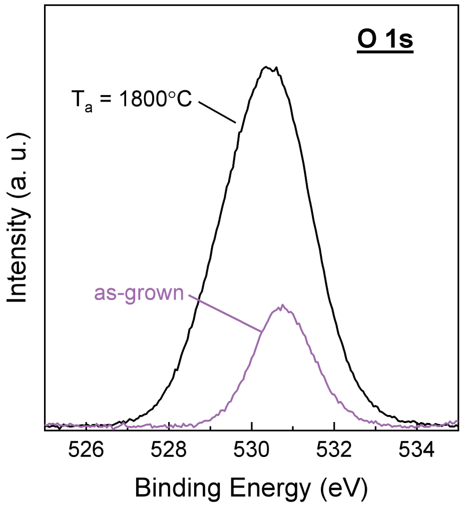

Supplementary Figure S11 | XPS O 1s spectra for hBN on the AlN/sapphire template before and after annealing at 1800 °C.

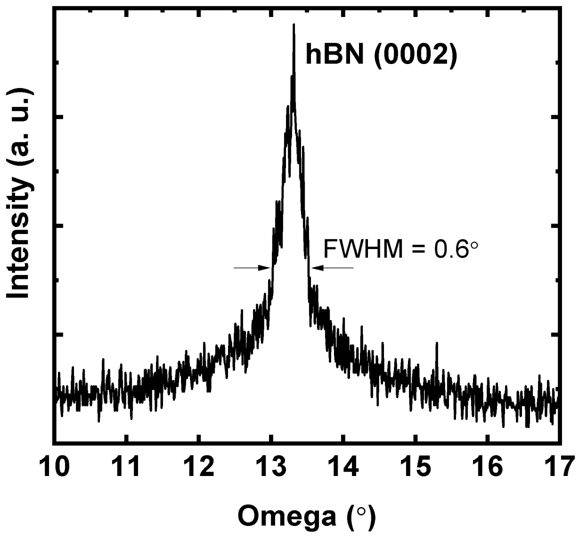

Supplementary Figure S12 | X-ray rocking curve of the hBN (0002) reflection for hBN on an AlN substrate after annealing at 1800 °C.

References

[1] Y. Hattori, T. Taniguchi, K. Watanabe, K. Nagashio, *ACS Nano* **2015**, *9*, 916.

[2] Y. Hattori, T. Taniguchi, K. Watanabe, K. Nagashio, *ACS Appl. Mater. Interfaces* **2016**, *8*, 27877.

[3] A. Ranjan, N. Raghavan, M. Holwill, K. Watanabe, T. Taniguchi, K. S. Novoselov, K. L. Pey, S. J. O'Shea, *ACS Appl. Electron. Mater.* **2021**, *3*, 3547.

[4] S. Hong, C.-S. Lee, M.-H. Lee, Y. Lee, K. Y. Ma, G. Kim, S. I. Yoon, K. Ihm, K.-J. Kim, T. J. Shin, S. W. Kim, E. Jeon, H. Jeon, J.-Y. Kim, H.-I. Lee, Z. Lee, A. Antidormi, S. Roche, M. Chhowalla, H.-J. Shin, H. S. Shin, *Nature* **2020**, *582*, 511.

[5] S. K. Jang, J. Youn, Y. J. Song, S. Lee, *Sci Rep* **2016**, *6*, 30449.

[6] A. Bansal, M. Hilse, B. Huet, K. Wang, A. Kozhakhmetov, J. H. Kim, S. Bachu, N. Alem, R. Collazo, J. A. Robinson, R. Engel-Herbert, J. M. Redwing, *ACS Appl. Mater. Interfaces* **2021**, *13*, 54516.

[7] A.-R. Jang, S. Hong, C. Hyun, S. I. Yoon, G. Kim, H. Y. Jeong, T. J. Shin, S. O. Park, K. Wong, S. K. Kwak, N. Park, K. Yu, E. Choi, A. Mishchenko, F. Withers, K. S. Novoselov, H. Lim, H. S. Shin, *Nano Lett.* **2016**, *16*, 3360.
